# Amygdala parcellation reveals unique subregions of atrophy in FTLD‐tauopathies

**DOI:** 10.1002/alz70862_109842

**Published:** 2025-12-23

**Authors:** Jane K Stocks, Rachel M Keszycki, Daniel Gutstein, Todd Parrish, Rudolph J Castellani, Changiz Geula, Elena Barbieri, Sandra Weintraub, Marsel Mesulam, Tamar Gefen

**Affiliations:** ^1^ Northwestern University Feinberg School of Medicine, Chicago, IL USA; ^2^ Brown University, Providence, RI USA; ^3^ Northwestern University, Chicago, IL USA; ^4^ Mesulam Center for Cognitive Neurology & Alzheimer's Disease, Chicago, IL USA

## Abstract

**Background:**

Primary progressive aphasia (PPA) is a dementia syndrome characterized by language decline that can be caused by frontotemporal lobar degeneration with tau pathology (FTLD‐tau), involving three or four microtubule binding repeat domains (i.e., 3R and 4R tau). Evidence suggests that differential atrophy in limbic regions such as the amygdala may play a role in the clinical heterogeneity of 3R and 4R FTLD‐tau. This study examined the distribution of atrophy across amygdala subregions in these tauopathies.

**Method:**

Eighteen right‐handed PPA cases and 35 age‐matched controls from the Northwestern PPA Program underwent structural MRI. PPA cases were chosen based on postmortem pathology of 3R (*N* = 9) or 4R (*N* = 9) FTLD‐tauopathy. T1‐weighted images were acquired on a Siemens 3T scanner and underwent FreeSurfer (v7) processing. Amygdala nuclei were parcellated using FreeSurfer’s amygdala subregion module and normalized to each individual’s estimated total intracranial volume. Mixed linear regressions, adjusting for disease duration and incorporating random intercepts, were used to compare amygdala subregion volumes between tauopathies and controls.

**Result:**

PPA/3R had a mean age of 63.5 years (SD = 3.9), PPA/4R were 72.5 years (SD = 5.8), and controls were 63.4 years (SD = 6.8). In the left hemisphere, PPA/3R cases had smaller mean volumes than controls in the whole amygdala (*p* = 0.001) and all nuclei except the medial and paralaminar nuclei (i.e., lateral, basal, accessory basal, anterior amygdaloid area, cortical, central, and corticoamygdaloid transition area, all *p* <0.05). PPA/4R cases demonstrated smaller volumes than controls in only the paralaminar (*p* = 0.03) and corticoamygdaloid transition area (*p* = 0.009). Compared to PPA/4R, PPA/3R cases had reduced volumes in the whole amygdala (*p* = 0.04), lateral (*p* = 0.04), and cortical (*p* = 0.03) nuclei. There were no significant differences between disease groups in the right hemisphere.

**Conclusion:**

Results highlight the leftward asymmetry of disease in PPA and demonstrate increased atrophy of the amygdala in PPA/3R. Further, distinct portions of the amygdala were implicated in 3R versus 4R FTLD‐ tauopathy. Findings support prior research showing divergent neuropsychiatric phenotypes in FTLD‐tauopathies, which may result from disruptions in amygdalar circuits governing emotional and behavioral processes. Future functional connectivity studies could offer greater insight into the pathways of limbic disruption in tauopathies.

